# Supplementary material for: A multi-country, multi-year, meta-analytic evaluation of the sex differences in age-specific pertussis incidence rates
Source: PLoS One. 2020 Apr 23;15(4):e0231570. doi: 10.1371/journal.pone.0231570 (PMC7179848; doi:10.1371/journal.pone.0231570)
Supplement: Attachment — Submitted filename: Response to Reviewers.docx.pdf [file pone.0231570.s003.pdf]

PONE-D-19-34094R1
A multi-country, multi-year, meta-analytic evaluation of the sex differences in age-specific pertussis incidence rates
PLOS ONE

Dear Dr Victoria Peer,

Thank you for submitting your manuscript to PLOS ONE. After careful consideration, we feel that it has merit but does not fully meet PLOS ONE’s publication criteria as it currently stands. Therefore, we invite you to submit a revised version of the manuscript that addresses the following minor point to ensure clarity of reporting. In the manuscript text, the authors frequently describe their study using the term 'meta-analysis', we suggest to replace this term by ' meta-analytic methods ' to describe their analyses.

Response:

Dear Dr.Daniela Flavia Hozbor,

We replaced the term 'meta-analysis' with term 'meta-analytic methods' throughout the article.

Reviewers' comments:

Reviewer's Responses to Questions

Comments to the Author

1. If the authors have adequately addressed your comments raised in a previous round of review and you feel that this manuscript is now acceptable for publication, you may indicate that here to bypass the “Comments to the Author” section, enter your conflict of interest statement in the “Confidential to Editor” section, and submit your "Accept" recommendation.

Reviewer #1: All comments have been addressed

---

2. Is the manuscript technically sound, and do the data support the conclusions?

The manuscript must describe a technically sound piece of scientific research with data that supports the conclusions. Experiments must have been conducted rigorously, with appropriate controls, replication, and sample sizes. The conclusions must be drawn appropriately based on the data presented.

Reviewer #1: Yes

---

3. Has the statistical analysis been performed appropriately and rigorously?

Reviewer #1: Yes

---

4. Have the authors made all data underlying the findings in their manuscript fully available?

The PLOS Data policy requires authors to make all data underlying the findings described in their manuscript fully available without restriction, with rare exception (please refer to the Data Availability Statement in the manuscript PDF file). The data should be provided as part of the manuscript or its supporting information, or deposited to a public repository. For example, in addition to summary statistics, the data points behind means, medians and variance measures should be available. If there are restrictions on publicly sharing data—e.g. participant privacy or use of data from a third party—those must be specified.

Reviewer #1: Yes

---

5. Is the manuscript presented in an intelligible fashion and written in standard English?

PLOS ONE does not copyedit accepted manuscripts, so the language in submitted articles must be clear, correct, and unambiguous. Any typographical or grammatical errors should be corrected at revision, so please note any specific errors here.

Reviewer #1: Yes

---

6. Review Comments to the Author

Please use the space provided to explain your answers to the questions above. You may also include additional comments for the author, including concerns about dual publication, research ethics, or publication ethics. (Please upload your review as an attachment if it exceeds 20,000 characters)

Reviewer #1: Re. comment 10. While it is true that maternal estrogens disappear from fetal circulation within a few weeks of birth the biological effects of the in utero estrogen exposure will linger for months. Hence my comment about the reduced M:F gradient among infants still stands as a plausible explanation. And as I stated before, I believe that this strengthens your argument.

Response: We completely agree with the reviewer and think that the reviewer's comment may be another possible mechanism and explanation for the results. We have now added that to the discussion on lines 289-295 in the revised manuscript.

---

7. PLOS authors have the option to publish the peer review history of their article (what does this mean?). If published, this will include your full peer review and any attached files.

If you choose “no”, your identity will remain anonymous but your review may still be made public.

Do you want your identity to be public for this peer review?
For information about this choice, including consent withdrawal, please see our Privacy Policy.

Reviewer #1: Yes: Christopher Gill

[NOTE: If reviewer comments were submitted as an attachment file, they will be attached to this email and accessible via the submission site. Please log into your account, locate the manuscript record, and check for the action link "View Attachments". If this link does not appear, there are no attachment files to be viewed.]
