# Supplementary material for: A multi-country, multi-year, meta-analytic evaluation of the sex differences in age-specific pertussis incidence rates
Source: PLoS One. 2020 Apr 23;15(4):e0231570. doi: 10.1371/journal.pone.0231570 (PMC7179848; doi:10.1371/journal.pone.0231570)
Supplement: Attachment — Submitted filename: Amendments.docx [file pone.0231570.s002.docx]

PONE-D-19-34094R1

A multi-country, multi-year, meta-analytic evaluation of the sex differences in age-specific pertussis incidence rates

Thank you for submitting your manuscript entitled "A multi-country, multi-year, meta-analytic evaluation of the sex differences in age-specific pertussis incidence rates" to PLOS ONE.  Your manuscript files have been checked in-house but before we can proceed we need you to address the following issues:

1) Please ensure that you refer to Figure 8 in your text as, if accepted, production will need this reference to link the reader to the figure.

*Answer: We referred to Figure 8 in the text*

2) Please amend your list of authors on the manuscript to ensure that each author is linked to an affiliation.

We note that you have included affiliation numbers 1,¶  and * however only affiliations 1 has authors linked to them.  Please amend affiliation 4 to link an author to it or remove if added in error.

*Answer: We amended the list of authors and affiliation on the title page*

3) Please allow me to provide more insight into how much of your data you are meant to publicly share and what exactly constitutes a minimal data set or underlying data.

We require authors to share the “minimal data set” for their submission. PLOS defines the minimal data set to consist of the data required to replicate all study findings reported in the article, as well as related metadata and methods. Additionally, PLOS requires that authors comply with field-specific standards for preparation, recording, and deposition of data when applicable. For example, authors should submit the following data:

1) The values behind the means, standard deviations and other measures reported;

2) The values used to build graphs;

3) The points extracted from images for analysis.

Authors do not need to submit their entire data set if only a portion of the data were used in the reported study. Also, authors do not need to submit the raw data collected during an investigation if the standard in the field is to share data that have been processed.

*Answer: All relevant data are within the manuscript.*

*Table number 1 contains all the minimal data used for the study ( for meta-analysis).*

*On this table we performed* ***all the data*** *(“minimal data set” for* ***all reported years*** *together) about pertussis cases by sex (****cumulative total*** ***n****), total population by sex (****cumulative total N****), calculated incidence rate per 100 000 male or female population, and incidence rate ratio (female: male incidence rate ratio).All this data required for study findings replication.*

*If needed we could share the* ***all raw data by single year and sex for each country*** *(pertussis cases by sex* ***n*** *by single year and total population by sex* ***N*** *by single year).*

Additionally, we require authors to provide sample image data in support of all reported results (e.g. for immunohistochemistry images, fMRI images, etc.), either with the submission files or in a public repository.

*Answer: We have no any other image data in support of our findings*

At this time, please confirm that the minimal data set are within your paper.

*Answer: We confirm that the minimal data set are within the paper.*
